# Social Media Use and Its Concurrent and Subsequent Relation to a Biological Marker of Inflammation: Short-Term Longitudinal Study

**DOI:** 10.2196/46309

**Published:** 2023-12-08

**Authors:** David Lee, Tao Jiang, Jennifer Crocker, Baldwin Way

**Affiliations:** 1 Department of Communication University at Buffalo, The State University of New York Buffalo, NY United States; 2 Institute for Policy Research Northwestern University Evanston, IL United States; 3 Department of Psychology The Ohio State University Columbus, OH United States

**Keywords:** social media use, inflammation, physical health, well-being, screen time, mental health, social media, biomarker, chronic disease

## Abstract

**Background:**

Although many studies have examined the impact of social media use (SMU) on mental health, very few studies have examined the association of SMU with health-relevant biomarkers.

**Objective:**

Addressing this gap, we conducted a short-term longitudinal study examining the link between SMU and C-reactive protein (CRP), a biological marker of systemic inflammation predictive of major depression, chronic diseases, and mortality.

**Methods:**

We measured college students’ weekly amount of SMU for 5 consecutive weeks objectively via the Screen Time app and collected blood samples at baseline and 5 weeks later.

**Results:**

In separate cross-sectional analyses conducted at phase 1 (baseline) and at phase 2 (5 weeks after baseline), objective SMU had a positive, concurrent association with CRP at both time points. Critically, in a longitudinal analysis, more SMU between phase 1 and phase 2 predicted increased CRP between these time points, suggesting that increased SMU led to heightened inflammation during that period.

**Conclusions:**

Although more research is needed to understand why SMU led to higher inflammation, the association between objective SMU and a marker of a biological process critical to physical health presents an intriguing opportunity for future research on social media effects.

## Introduction

### Background

The past decade has witnessed a plethora of studies examining the impact of social media use (SMU) on daily lives [1,2]. For example, more than 80 meta-analyses and reviews have examined the impact of SMU on psychological well-being and mental health [3]. By comparison, however, much less work has investigated the impact of SMU on health-relevant biology. This is surprising given the importance of physical health and the recent public dialog on the potentially harmful effects of SMU [4]. The goal of this research is to begin addressing this knowledge gap. Specifically, we examine how SMU is associated with a biological process that influences physical health, namely, systemic inflammation—a potent driver of chronic illnesses such as cardiovascular diseases, type 2 diabetes, and multiple cancers [5].

### Physical Health Implications of Inflammation

Inflammation, a form of activation of the immune system, is a key biological process that affects physical health [6]. While acute, local inflammation promotes healing by facilitating the elimination of viruses and pathogens, chronic, systemic, and low-grade inflammation may have detrimental health consequences by affecting many health-relevant systems in the body [7], including the brain [8]. While multiple biomarkers in the blood have been used to assess inflammation, one of the most commonly used biomarkers is C-reactive protein (CRP). Elevated CRP is associated with an increased risk of cardiovascular disease [9] and can predict multiple forms of cancer, including lung, breast, and prostate cancers [10]; type 2 diabetes [11]; and earlier mortality [12]. Thus, the broad health consequences of inflammation suggest that it is an important biological pathway that can influence physical health.

### SMU and Inflammation

How might SMU influence inflammation? Several perspectives suggest a potential link. One possibility is that SMU may influence health-related behaviors. For instance, some studies show that excessive SMU or screen time may undermine the quality and quantity of sleep [13,14]. Poor quality or insufficient amount of sleep is linked to higher inflammation [15,16]. Similarly, prolonged SMU may contribute to sedentary lifestyles and physical inactivity [17,18], which can increase inflammation levels [19,20]. This perspective is consistent with the displacement hypothesis, which argues that SMU may have a negative impact on individuals if the time spent using it displaces health-promoting activities [21,22].

Another possibility is that SMU may increase stress, which can elevate inflammation [5,23,24]. For example, scholars have argued that hyperconnectivity—the permanent availability of and connectivity to other people and various media contents on social media—can heighten stress [14,25,26]. Moreover, on social media, people may encounter information that can contribute to stress. For example, people may be exposed to offensive or hateful content and experience hate on the web [27,28]. Other times, they may come across other people’s achievements and positive news on social media [2]; constant exposure to such information about others can trigger upward social comparisons and feelings of envy [29]. Such experience of stress, whether real or imagined, can trigger proinflammatory responses [30].

Consistent with the above perspectives, recent cross-sectional studies have provided initial evidence for the link between the amount of SMU and inflammation. For example, in one of the first studies on the relation between a biological marker of inflammation and technology use, Afifi et al [23] discovered a positive correlation between adolescent’s self-reported Facebook use and salivary interleukin-6 (an inflammatory biomarker that triggers CRP synthesis [31]). In another cross-sectional study, college students’ self-reported SMU across 4 platforms (ie, Snapchat, Instagram, Facebook, and Twitter) was positively associated with CRP in the blood [32].

Although these initial studies begin to suggest an impact of SMU on inflammation, they also have some limitations. First, these studies measured the amount of SMU by asking participants to retrospectively recall their amount of use over several days, a methodological approach that has been shown to provide imprecise estimates [33]. For instance, one study of approximately 50,000 people found that various survey measures of Facebook behavior correlated only moderately (0.23<*r*<0.42) with people’s actual Facebook use [34]. Thus, it is unclear how precisely the amount of SMU is associated with inflammation. Second, these studies’ reliance on cross-sectional data limits the ability to draw strong temporal or causal interpretations, which has been noted as a “major weakness” in social media research [3,35]. Thus, this research sought to address these issues by measuring SMU objectively via the Screen Time app and using a short-term longitudinal design. Specifically, we tested the following hypotheses:

- H1: Higher SMU, measured objectively, will be associated with higher levels of inflammation (CRP) concurrently.
- H2: Higher SMU, measured objectively, will be associated with increased levels of inflammation (CRP) over time.

## Methods

### Participants and Procedure

Data collection for this study occurred between September 2021 and May 2022. We recruited 171 undergraduate students from a large Midwestern university in the United States. This longitudinal study consisted of 2 parts: a baseline laboratory session (phase 1) and a follow-up laboratory session after approximately 5 weeks (mean 38.69, SD 7.08 days; phase 2; n=140). Between phase 1 and phase 2, participants were sent 4 weekly surveys that prompted them to report their weekly SMU recorded by their iPhone’s iOS Screen Time app. Because the Screen Time app was only offered on the iOS operating system, our analyses were limited to 138 iPhone users for phase 1 (n=87, 63% female; n=48, 34.8% male; n=2, 1.4% others; n=1, 0.7% unreported; age: mean 19.13, SD 2.67; range 18-44 years; African American: n=16, 11.6%; Asian or Pacific Islander: n=34, 24.6%; Hispanic or Latin American: n=8, 5.8%; White: n=89, 64.5%; others: n=9, 6.5%) and 114 iPhone users for phase 2 (n=72, 63.2% female; n=40, 35.1% male; n=1, 0.9% other; n=1, 0.9% unreported; age: mean 19.10, SD 2.86; range 18-44 years; African American: n=14, 12.3%; Asian or Pacific Islander: n=23, 20.2%; Hispanic or Latin American: n=7, 6.1%; White: n=76, 66.7%; others: n=8, 7%).

During phase 1, participants completed several background questionnaires assessing factors such as sociodemographic information, SMU, health behaviors, and other measures not relevant to this investigation. Once participants completed the questionnaires, a trained research assistant collected their blood samples in a separate room. Participants could choose not to provide their blood and continue the study without losing compensation. In phase 1, a total of 14 (8.2%) out of 171 participants opted out of the blood sample collection procedure, and another 4 (2.3%) participants provided insufficient amount of blood to assay. Among these 18 participants, only 13 participants were removed from the total sample because the other 5 participants had already been removed for being non-iPhone users. Approximately 5 weeks after completing phase 1, participants returned to the laboratory for phase 2. We decided on a 5-week window between phase 1 and phase 2 because CRP has been shown to vary over comparable time periods [36-38]. Samples were collected throughout the day due to a lack of diurnal variability in CRP [39,40]. Similar to phase 1, in phase 2, participants completed various questionnaires and provided blood samples for CRP. In phase 2, a total of 14 (10%) out of 140 participants opted out of the blood collection procedure, and another 36 (25.7%) participants did not provide their blood samples because their data collection occurred when we had paused the collection of blood samples due to an institutional review board related issue. Among these 50 participants, only 40 participants were removed from the total sample because the other 10 participants had already been removed for being non-iPhone users. Finally, an additional 10 participants from phase 1 and 8 participants from phase 2 were removed from the analysis because they did not have the Screen Time app enabled over the course of data collection and therefore did not have reliable screen time information available. Thus, the final sample available for analyses for this study involved 115 participants for phase 1 and 66 participants for phase 2.

### Measures

#### Phases 1 and 2 CRP

CRP was assayed from dried blood spots following an established protocol from prior work [41]. First, we swabbed participants’ fingers with alcohol and pricked them with an 18-gauge needle (Unistick 3; Owen Mumford). The blood drops were collected on a 903 Protein Saver Card (Whatman) and kept for 24 hours to dry at room temperature. Next, we punched the samples with a 3-mm punch and stored them in microcentrifuge tubes at –80 °C. To assay for CRP, we thawed a single 3-mm punch and added 200 µL of buffer (phosphate buffered saline with 0.1% Tween 20; ThermoFisher Scientific) overnight incubation at 4 °C while shaking at 60 rpm. We diluted this eluate 1:10 and assayed CRP the following morning using Vplex Plus kits (K151STG; Meso Scale Delivery). All samples were successfully measured (ie, within the linear range of the standard curve). The intraassay coefficient of variation was 3.8%, while the interassay coefficient of variation was 11.04% (phase 1: mean 0.99, SD 1.75 mg/L and phase 2: mean 0.98, SD 1.48 mg/L).

#### SMU Measurement

Participants reported the amount of time they spent using each of the 4 social media platforms (ie, Snapchat, Instagram, Twitter, and Facebook) every week between the weeks of phase 1 and phase 2 (across 5 weeks). At the phase 1 session, participants were instructed on how to retrieve this information from the iOS Screen Time app on their iPhone (see Multimedia Appendix 1 for details).

We measured SMU across 4 platforms for the following reasons. First, at the time of our study design, Snapchat, Instagram, Twitter, and Facebook were the most commonly used social media platforms among college students [42]. Second, recent work suggests collecting SMU across multiple platforms because most people use multiple platforms in varying amounts [43]. Third, this approach is consistent with our prior work using self-reported SMU [32].

To examine the cross-sectional (ie, concurrent) relations between SMU and CRP, 2 separate predictors were created for SMU (ie, phase 1 SMU and phase 2 SMU). For phase 1 SMU, we summed participants’ SMU across the 4 platforms during the week when participants visited our laboratory initially to provide their blood samples (mean 579.05, SD 436.70 min). More specifically, the Screen Time app records SMU for the entire week from one Sunday to the next Sunday. The blood samples were collected on weekdays. To obtain overlap between the SMU measure and the blood measure, we used the SMU value from the first SMU survey sent to participants on the first Monday after the blood draw, which actually reflects SMU over the week in which the blood was sampled. For phase 2 SMU, because the phase 2 laboratory session marked the last day of study participation for each participant, SMU data over the week in which the phase 2 blood was drawn were not available. Thus, we collected the amount of SMU for the week preceding the week when participants visited our laboratory the second time to provide blood (mean 615.84, SD 395.87 min). Although this variable does not precisely overlap with phase 2 CRP measurement, on average, phase 2 SMU preceded phase 2 CRP by 2.9 (SD 1.46) days.

Finally, to examine the potential longitudinal impact of SMU on CRP, we created another predictor by computing the weekly average of the total amount of time spent on social media from phase 1 to phase 2, which spanned 5 weeks (mean 587.58, SD 387.04 min). This variable (ie, phase 1 to 2 SMU) allowed us to test whether SMU from phase 1 to phase 2 predicted changes in CRP between phase 1 and phase 2.

#### Covariates

Consistent with recent recommendations [44] and prior work [32], we controlled for additional variables that can influence inflammation. Specifically, sociodemographic covariates were included (ie, sex, age, household income, and the highest level of education completed by mother and father; 1=some high school and 5=graduate school). We also controlled for health-related behaviors such as BMI, cigarette smoking (ie, number of days participants smoked cigarettes in the past 30 days; 1=0 day, 2=1 or 2 days, 3=3 to 5 days...7=20 to 29 days, and 8=all 30 days), frequency of alcohol consumption (1=never, 2=several times a year, 3=monthly, 4=2-4 times a month, 5=2-3 times a week, and 6=4 or more times a week), and amount of time spent sitting in the past month (1=none, 2=a little, 3=a moderate amount, 4=a lot, and 5=a great deal). Additional covariates included depressive symptoms (Center for Epidemiological Studies Depression Scale [45]) and the use of birth control pills (0=no and 1=yes; n=42) because they can influence inflammation [44].

### Ethical Considerations

The institutional review board at the Ohio State University (protocol 2018H0452) approved this study. Informed consent was obtained from all participants. Data from this study were deidentified prior to analyses. Participants received partial course credit as compensation.

## Results

### Data Cleaning and Exclusions

Consistent with conventional approaches of analyzing CRP, 1 participant whose CRP value was greater than 10 µg/mL was removed from analyses because such values are likely to indicate an acute infection rather than heightened inflammation due to psychosocial factors such as SMU [46]. Including this participant in the analyses did not change any results substantively. Table 1 includes zero-order correlations for all key variables.

**Table 1:** Zero-order correlations among key variables.

Variables			SMU (P1)^a^		SMU (P2)^b^		Avg SMU (P1-P2)^c^		CRP (P1)^d^		CRP (P2)^e^		Sex^f^		Age		BMI		Depressive symptoms
**SMU (P1)**																			
	*r*	—^g^		0.75		0.91		0.23		0.36		0.14		–0.19		–0.07		–0.01	
	*P* value	—		<.001		<.001		.02		.005		.17		.06		.51		.95	
	n^h^	104		90		104		104		59		103		103		104		104	
**SMU (P2)**																			
	*r*	0.75		—		0.88		0.25		0.37		0.08		–0.19		–0.01		–0.14	
	*P* value	<.001		—		<.001		.02		.004		.44		.07		.90		.19	
	n	90		92		92		92		61		91		91		92		91	
**Avg SMU (P1-P2)**																			
	*r*	0.91		0.88		—		0.21		0.37		0.18		–0.20		–0.01		–0.01	
	*P* value	<.001		<.001		—		.03		.003		.07		.04		.90		.99	
	n	104		92		109		109		61		108		108		109		107	
**CRP (P1)**																			
	*r*	0.23		0.25		0.21		—		0.77		0.10		0.02		0.37		0.11	
	*P* value	.02		.02		.03		—		<.001		.30		.87		<.001		.27	
	n	104		92		109		114		62		113		113		113		108	
**CRP (P2)**																			
	*r*	0.36		0.37		0.37		0.77		—		0.27		0.02		0.35		–0.01	
	*P* value	.005		.004		.003		<.001		—		.03		.88		.005		.96	
	n	59		61		61		62		62		61		61		62		61	
**Sex**																			
	*r*	0.14		0.08		0.18		0.10		0.27		—		–0.08		–0.05		0.15	
	*P* value	.17		.44		.07		.30		.03		—		.41		.61		.14	
	n	103		91		108		113		61		61		113		112		107	
**Age**																			
	*r*	–0.19		–0.19		–0.20		0.02		0.02		–0.08		—		0.03		–0.05	
	*P* value	.06		.07		.04		.87		.88		.41		—		.75		.63	
	n	103		91		108		113		61		113		113		112		107	
**BMI**																			
	*r*	–0.07		–0.01		–0.01		0.37		0.35		–0.05		0.03		—		0.04	
	*P* value	.51		.90		.90		<.001		.005		.61		.75		—		.69	
	n	104		92		109		113		62		112		112		113		108	
**Depressive symptoms**																			
	*r*	–0.01		–0.14		–0.01		0.11		–0.01		0.15		–0.05		0.04		—	
	*P* value	.95		.19		.99		.27		.96		.14		.63		.69		—	
	n	104		91		107		108		61		107		107		108		108	

^a^SMU (P1): social media use at phase 1.

^b^SMU (P2): social media use at phase 2.

^c^Avg SMU (P1-P2): average weekly social media use from phase 1 to phase 2.

^d^CRP (P1): logged C-reactive protein at phase 1.

^e^CRP (P2): logged C-reactive protein at phase 2.

^f^Sex: coded with 1 (male) and 2 (female).

^g^Not available.

^h^n values vary due to missing values.

### Testing H1: Is SMU Positively Associated With CRP Concurrently?

**Figure 1:**
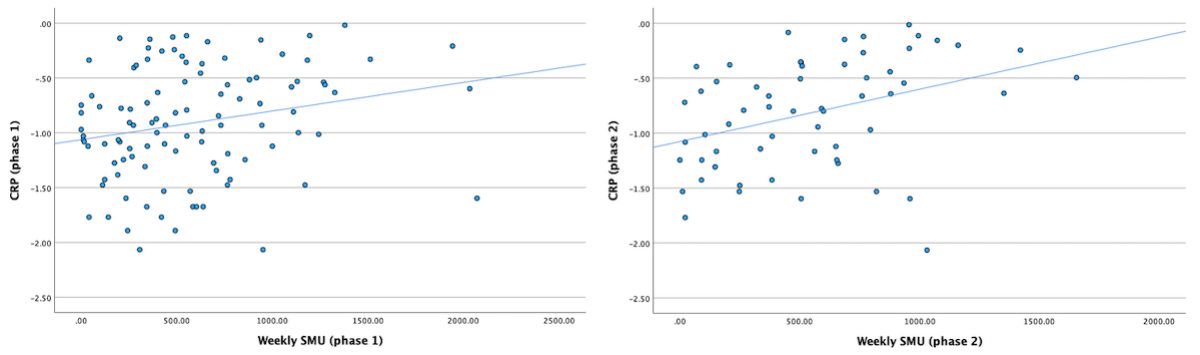
Scatterplots of the cross-sectional relation between SMU and CRP. CRP: C-reactive protein; SMU: social media use.

We conducted 2 sets of multiple regression analyses to test the cross-sectional relations between SMU and CRP (phases 1 and 2). Consistent with the approach taken in our prior work on SMU and CRP [32], the models sequentially controlled for an increasing number of covariates to provide details on how the association between SMU and CRP is influenced by the covariates: (1) sociodemographic factors, (2) health-related behaviors, (3) depressive symptoms, and (4) the use of birth control. Consistent with our hypothesis, SMU at phase 1 was positively associated with CRP levels at phase 1 in model 1 (β=.25; *P*=.02), model 2 (β=.22; *P*=.02), model 3 (β=.23; *P*=.03), and model 4 (β=.24; *P*=.01). Similarly, SMU at phase 2 was positively associated with CRP levels at phase 2 in model 1 (β=.36; *P*=.01), model 2 (β=.41; *P*=.005), model 3 (β=.39; *P*=.007), and model 4 (β=.37; *P*=.01). Thus, H1 was supported. The results of these analyses are summarized in Tables 2 and 3, where we report unstandardized coefficients to supplement the standardized coefficients reported here in the text. Figure 1 depicts scatterplots of the correlation between SMU and CRP at phase 1 (left panel) as well as the correlation between SMU and CRP at phase 2 (right panel) without any covariates.

**Table 2:** Coefficients from linear regression models predicting C-reactive protein at phase 1 (n=102 due to missing values).

Predictor	Model 1					Model 2					Model 3					Model 4			
	β^a^	SE	*t* test (*df*)	*P* value	β		SE	*t* test (*df*)	*P* value	β		SE	*t* test (*df*)	*P* value	β		SE	*t* test (*df*)	*P* value
Sex^b^	.12	0.11	1.11 (96)	.27	.18		0.11	1.66 (92)	.10	.17		0.11	1.61 (91)	.11	–.02		0.12	–0.20 (90)	.84
Age	.02	0.02	1.13 (96)	.26	.02		0.02	1.23 (92)	.22	.02		0.02	1.37 (91)	.17	.02		0.02	0.95 (90)	.34
Edu (M)^c^	–.06	0.06	–0.99 (96)	.33	–.01		0.06	–0.04 (92)	.97	.01		0.06	0.18 (91)	.86	–.02		0.06	–0.32 (90)	.75
Edu (F)^d^	.04	0.05	0.81 (96)	.42	.02		0.05	0.41 (92)	.69	.02		0.05	0.38 (91)	.70	.04		0.04	0.88 (90)	.38
Income^e^	.03	0.02	1.27 (96)	.21	.03		0.02	1.36 (92)	.18	.03		0.02	1.35 (91)	.18	.02		0.02	0.86 (90)	.39
BMI	N/A^f^	N/A	N/A	N/A	.05		0.01	4.67 (92)	<.001	.05		0.01	4.63 (91)	<.001	.05		0.01	4.73 (90)	<.001
Smoking^g^	N/A	N/A	N/A	N/A	–.03		0.04	–0.75 (92)	.45	–.03		0.04	–0.73 (91)	.47	–.02		0.04	–0.57 (90)	.57
Alcohol^h^	N/A	N/A	N/A	N/A	.06		0.03	1.83 (92)	.07	.06		0.03	1.76 (91)	.08	.05		0.03	1.44 (90)	.15
Sit^i^	N/A	N/A	N/A	N/A	–.05		0.06	–0.84 (92)	.40	–.08		0.06	–1.26 (91)	.21	–.05		0.06	–0.74 (90)	.46
Depressive symptoms	N/A	N/A	N/A	N/A	N/A		N/A	N/A	N/A	.12		0.09	1.36 (91)	.18	.09		0.09	1.06 (90)	.29
BirthCon^j^	N/A	N/A	N/A	N/A	N/A		N/A	N/A	N/A	N/A		N/A	N/A	N/A	.33		0.11	–2.93 (90)	.004
SMU P1^k^	.01	0.01	2.41 (96)	.02	.01		0.01	2.32 (92)	.02	.01		0.01	2.28 (91)	.03	.01		0.01	2.59 (90)	.01
*R* ^2^ ^l^	0.09					0.29					0.30					0.36			

^a^β values reflect unstandardized coefficients*.*

^b^Sex: coded with 1 (male) and 2 (female).

^c^Edu (M): highest degree obtained by mother.

^d^Edu (F): highest degree obtained by father.

^e^Income: family annual income.

^f^N/A: not applicable.

^g^Smoking: cigarettes smoked per day in the last 30 days.

^h^Alcohol: frequency of alcohol consumption.

^i^Sit: amount of time spent sitting in the past month.

^j^BirthCon: consumption of birth control medication. BirthCon was coded with 0 (not currently taking birth control medication) and 1 (currently taking birth control medication).

^k^SMU P1: social media use at phase 1.

^l^*R*^2^ values reflect those with social media use in the models.

**Table 3:** Coefficients from linear regression models predicting C-reactive protein at phase 2 (n=51 due to missing values).

Predictor	Model 1					Model 2					Model 3					Model 4			
	β^a^	SE	*t* test (*df*)	*P* value	β		SE	*t* test (*df*)	*P* value	β		SE	*t* test (*df*)	*P* value	β		SE	*t* test (*df*)	*P* value
Sex^b^	.25	0.15	1.73 (45)	.09	.20		0.14	1.42 (41)	.16	.19		0.14	1.39 (40)	.17	.02		0.18	0.09 (39)	.93
Age	.04	0.02	1.94 (45)	.06	.04		0.02	2.00 (41)	.05	.04		0.02	2.11 (40)	.04	.03		0.02	1.80 (39)	.08
Edu (M)^c^	–.16	0.08	–2.12 (45)	.04	–.13		0.08	–1.54 (41)	.13	–.11		0.08	–1.36 (40)	.18	–.12		0.08	–1.44 (39)	.16
Edu (F)^d^	.05	0.07	.69 (45)	.50	.03		0.07	0.39 (41)	.70	.04		0.07	0.57 (40)	.57	.05		0.07	0.73 (39)	.47
Income^e^	.06	0.03	1.95 (45)	.06	.06		0.03	1.97 (41)	.06	.06		0.03	1.80 (40)	.08	.05		0.03	1.56 (39)	.13
BMI	N/A^f^	N/A	N/A	N/A	.03		0.01	2.34 (41)	.02	.03		0.01	2.40 (40)	.02	.03		0.01	2.66 (39)	.01
Smoking^g^	N/A	N/A	N/A	N/A	–.05		0.09	–0.55 (41)	.59	–.05		0.09	–0.53 (40)	.60	–.03		0.08	–0.31 (39)	.76
Alcohol^h^	N/A	N/A	N/A	N/A	.09		0.05	1.87 (41)	.07	.08		0.05	1.82 (40)	.08	.07		0.05	1.46 (39)	.15
Sit^i^	N/A	N/A	N/A	N/A	.04		0.08	0.45 (41)	.65	.01		0.08	0.16 (40)	.87	.02		0.08	0.22 (39)	.83
Depressive symptoms	N/A	N/A	N/A	N/A	N/A		N/A	N/A	N/A	.12		0.10	1.15 (40)	.26	.13		0.10	1.23 (39)	.23
BirthCon^j^	N/A	N/A	N/A	N/A	N/A		N/A	N/A	N/A	N/A		N/A	N/A	N/A	–.26		0.17	–1.49 (39)	.14
SMU P2^k^	.01	0.01	2.56 (45)	.01	.01		0.01	2.97 (41)	.005	.01		0.01	2.82 (40)	.007	.01		0.01	2.68 (39)	.01
*R* ^2^ ^l^	0.26					0.41					0.43					0.46			

^a^β values reflect unstandardized coefficients*.*

^b^Sex: coded with 1 (male) and 2 (female).

^c^Edu (M): highest degree obtained by mother.

^d^Edu (F): highest degree obtained by father.

^e^Income: family annual income.

^f^N/A: not applicable.

^g^Smoking: cigarettes smoked per day in the last 30 days.

^h^Alcohol: frequency of alcohol consumption.

^i^Sit: amount of time spent sitting in the past month.

^j^BirthCon: consumption of birth control medication. BirthCon was coded with 0 (not currently taking birth control medication) and 1 (currently taking birth control medication).

^k^SMU P2: social media use at phase 2.

^l^*R*^2^ values reflect those with social media use in the models.

### Testing H2: Does SMU Over 5 weeks Predict Increased CRP?

**Figure 2:**
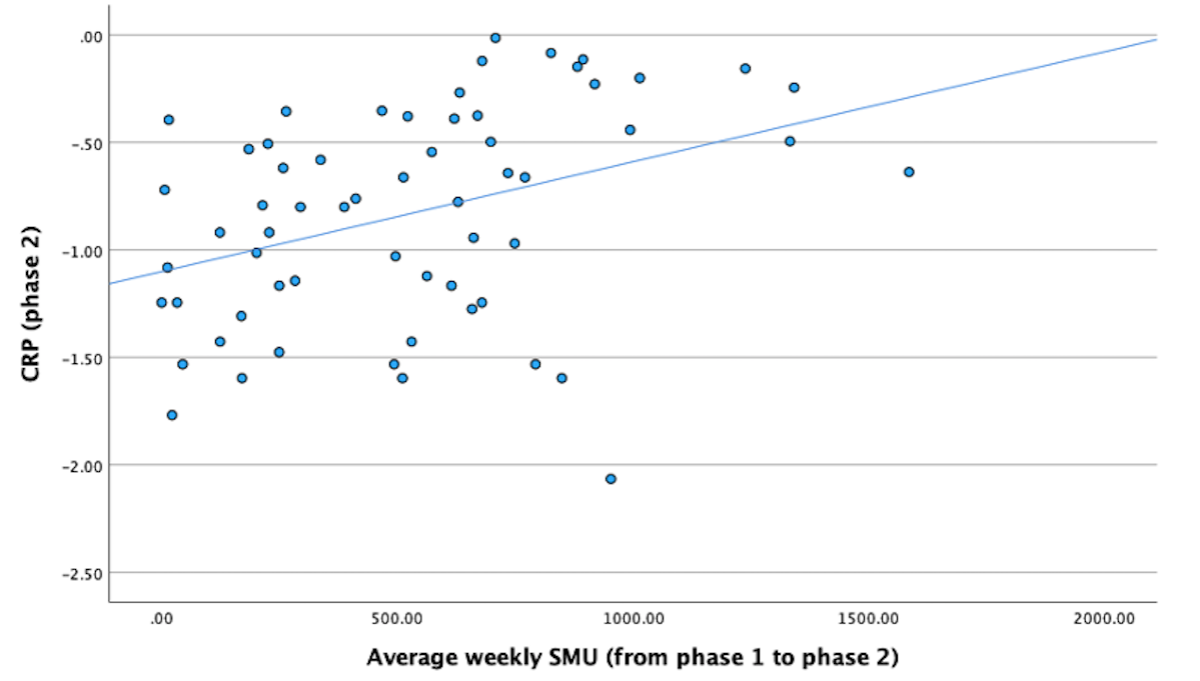
Scatterplot of the relation between weekly average SMU (from phase 1 to phase 2) and CRP (phase 2). CRP: C-reactive protein; SMU: social media use.

We conducted a series of multiple regression analyses with the amount of SMU between phase 1 and phase 2 as the independent variable while controlling for CRP at phase 1. The amount of SMU between phase 1 and phase 2 predicted changes in CRP from phase 1 to phase 2 in model 1 (β=.22; *P*=.04), model 2 (β=.28; *P*=.02), model 3 (β=.28; *P*=.02), and model 4 (β=.28; *P*=.02), suggesting that the impact of SMU on CRP may occur over time. Thus, H2 was also supported. The results of these analyses are detailed in Table 4. Figure 2 depicts a scatterplot of the correlation between weekly average SMU from phase 1 to phase 2 and CRP (phase 2) with no covariates. Supplementing these results, the amount of SMU at phase 1 also predicted CRP at phase 2 (controlling for CRP at phase 1) in model 1 (β=.28; *P*=.007), model 2 (β=.30; *P*=.005), model 3 (β=.31; *P*=.006), and model 4 (β=.30; *P*=.008). See Multimedia Appendix 1 for details of these results.

**Table 4:** Coefficients from the longitudinal linear regression model predicting C-reactive protein at phase 2 (n=53 due to missing values).

Predictor	Model 1					Model 2					Model 3					Model 4			
	β^a^	SE	*t* test (*df*)	*P* value	β		SE	*t* test (*df*)	*P* value	β		SE	*t* test (*df*)	*P* value	β		SE	*t* test (*df*)	*P* value
Sex^b^	.03	0.11	0.28 (45)	.79	.05		0.11	0.20 (41)	.68	.04		0.11	0.39 (40)	.70	.04		0.14	0.29 (39)	.77
Age	.02	0.01	1.56 (45)	.13	.02		0.01	1.61 (41)	.12	.02		0.01	1.51 (40)	.14	.02		0.01	1.48 (39)	.15
Edu (M)^c^	–.07	0.05	–1.32 (45)	.19	–.08		0.06	–1.31 (41)	.20	–.08		0.06	–1.30 (40)	.20	–.08		0.06	–1.27 (39)	.21
Edu (F)^d^	.03	0.05	0.69 (45)	.49	.03		0.05	0.56 (41)	.58	.03		0.05	0.53 (40)	.60	.03		0.05	0.52 (39)	.61
Income^e^	.02	0.02	0.67 (45)	.50	.02		0.02	0.86 (41)	.39	.02		0.02	0.84 (40)	.41	.02		0.02	0.83 (39)	.41
CRP P1^f^	.71	0.11	6.58 (45)	<.001	.62		0.13	4.84 (41)	<.001	.63		0.14	4.59 (40)	<.001	.63		0.14	4.38 (39)	<.001
Time^g^	.01	0.01	0.68 (45)	.50	.01		0.01	1.11 (41)	.29	.01		0.01	1.07 (40)	.29	.01		0.01	1.05 (39)	.30
BMI	N/A^h^	N/A	N/A	N/A	.01		0.01	0.73 (41)	.47	.01		0.01	0.68 (40)	.50	.01		0.01	0.66 (39)	.51
Smoking^i^	N/A	N/A	N/A	N/A	.01		0.06	0.09 (41)	.93	.01		0.06	0.09 (40)	.93	.01		0.06	0.09 (39)	.93
Alcohol^j^	N/A	N/A	N/A	N/A	.04		0.04	1.01 (41)	.32	.04		0.04	0.99 (40)	.33	.04		0.04	0.96 (39)	.34
Sit^k^	N/A	N/A	N/A	N/A	.06		0.06	0.99 (41)	.33	.07		0.07	1.00 (40)	.32	.07		0.07	1.00 (39)	.33
Depressive symptoms	N/A	N/A	N/A	N/A	N/A		N/A	N/A	N/A	–.02		0.08	–0.25 (40)	.80	–.02		0.09	–0.23 (39)	.82
BirthCon^l^	N/A	N/A	N/A	N/A	N/A		N/A	N/A	N/A	N/A		N/A	N/A	N/A	–.01		0.14	–0.03 (39)	.98
Avg SMU^m^	.01	0.01	2.15 (45)	.04	.01		0.01	2.52 (41)	.02	.01		0.01	2.50 (40)	.02	.01		0.01	2.42 (39)	.02
*R* ^2^ ^n^	0.65					0.67					0.67					0.67			

^a^β values reflect unstandardized coefficients*.*

^b^Sex: coded with 1 (male) and 2 (female).

^c^Edu (M): highest degree obtained by mother.

^d^Edu (F): highest degree obtained by father.

^e^Income: family annual income.

^f^CRP P1: C-reactive protein at phase 1.

^g^Time: days between C-reactive protein at phase 1 and C-reactive protein at phase 2.

^h^N/A: not applicable.

^i^Smoking: cigarettes smoked per day in the last 30 days.

^j^Alcohol: frequency of alcohol consumption.

^k^Sit: amount of time spent sitting in the past month.

^l^BirthCon: consumption of birth control medication. BirthCon was coded with 0 (not currently taking birth control medication) and 1 (currently taking birth control medication).

^m^Avg SMU: weekly average social media use from phase 1 to phase 2 over 5 weeks.

^n^*R*^2^ values reflect those with social media use in the models.

## Discussion

### Principal Findings

This research investigated how SMU is associated with CRP—a biological marker of inflammation linked with chronic illnesses such as cardiovascular disease and cancers. The results showed that the amount of SMU—assessed objectively via the Screen Time app—was not only associated with higher inflammation cross-sectionally but also an increase in inflammation 5 weeks later. The findings were consistent across different models adjusting for various covariates.

To our knowledge, this is one of the first studies to demonstrate a link between objective SMU across several platforms and CRP, a biomarker of inflammation. Building on prior work that found a positive correlation between self-reported SMU and inflammation [23,32], our longitudinal findings provide initial temporal evidence that SMU can lead to heightened inflammation. Importantly, that SMU predicted increased inflammation even after controlling for depressive symptoms is noteworthy because it suggests that the impact of SMU may extend beyond psychological well-being and that our results are not solely due to the depressogenic effects of inflammation [47].

Critically, the use of a health-relevant biomarker and the collection of objective SMU data across multiple platforms are key methodological strengths of this study. Compared with most prior studies that relied exclusively on self-report measures, our methodological approach is robust against survey response biases. Given these strengths, we encourage future research to use biological markers related to health or well-being and objective SMU data when applicable.

### Caveats and Limitations

There are some limitations of this study. First, this study tested an aggregate association between the amount of SMU across different platforms and inflammation. As an initial attempt to understand the potential link between SMU and inflammation, our goal was necessarily broad, focusing on the general metric of SMU amount—one of the most commonly measured and discussed variables in SMU research. Although this approach allowed us to better connect to extant research and public discourse (eg, SMU and well-being), fully understanding social media effects requires measuring processes much more nuanced and complex. Given that people use social media for different purposes (eg, entertainment, following the news, browsing, and supporting friends), future research should examine the different ways of using social media to illuminate what aspects of SMU are associated with inflammation. Relatedly, this research did not collect any data on the types of content people viewed on social media. Because these contents can drastically influence users' psychological experience [48], we cannot ascertain the extent to which the contents people interacted with contributed to our findings. Thus, future research should aim to collect data on the contents people view on social media to better capture their experience [49,50]. Moreover, future research should seek to understand specific mediating mechanisms (eg, sleep quality and stress) for our findings.

Another limitation of this work is that CRP was only measured twice over 5 weeks with a relatively small sample size. Although, to our knowledge, we have provided the first short-term longitudinal evidence for the impact of SMU on inflammation, our design does not allow for more sophisticated statistical models (eg, random intercept cross-lagged panel model) that can capture stability and intraindividual variability over time, for instance. Thus, future research should consider using high-powered intensive longitudinal designs with more frequent measurements of key variables and longer intervals. Alternatively, future research may consider manipulating people’s SMU and examine the potential causal effect of SMU on systemic inflammation. In addition, it is important to acknowledge that our sample did not include social media users who access social media through smartphones other than the iPhone or devices such as tablets and PCs. Thus, our SMU measure may not provide a comprehensive representation of SMU via different devices or operating systems. Future research should seek to replicate our findings with a larger sample from more diverse populations and contexts (eg, Android users and tablet users) to provide more confidence in the generalizability of the results. Relatedly, because social media effects are likely to vary at the individual level [3,48,51], future research may explore individual difference variables (eg, personality and loneliness) that may moderate our findings.

Finally, we note that the effect sizes observed in this study may be considered small to medium in size (from β=.19 to β=.41), which are comparable to (or even larger than) those typically found in studies on SMU and mental health (from *r*=–0.05 to *r*=–0.15). As several scholars have argued, small effect sizes can still have a meaningful impact [52-54], especially when considered at scale and over time [53,55]. We believe that this is especially likely to be the case for examining the potential biological impact of SMU among millions of young adults who are using social media for multiple hours every day for many years.

### Conclusions

This research discovered that objective SMU is positively associated with inflammation cross-sectionally and increased inflammation over time. The relation between SMU and inflammation presents an intriguing opportunity for future research that integrates social media effects and biological processes. Given the prevalence of SMU in the daily lives of adolescents and young adults and the societal importance of good physical health, more research investigating the potential physical health effects of SMU using diverse methodologies is needed.

## Appendix

Multimedia Appendix 1 Social media use data collection using the iOS Screen Time app.

## Abbreviations

CRP: C-reactive protein
SMU: social media use

## Data Availability

The data sets generated during and analyzed during this study are not publicly available due to sensitive health information content but are available from the corresponding author on reasonable request.
