# Correction: Social Media Use and Its Concurrent and Subsequent Relation to a Biological Marker of Inflammation: Short-Term Longitudinal Study

**DOI:** 10.2196/56808

**Published:** 2024-02-01

**Authors:** David Lee, Tao Jiang, Jennifer Crocker, Baldwin Way

**Affiliations:** 1 Department of Communication University at Buffalo, The State University of New York Buffalo, NY United States; 2 Institute for Policy Research Northwestern University Evanston, IL United States; 3 Department of Psychology The Ohio State University Columbus, OH United States

In “Social Media Use and Its Concurrent and Subsequent Relation to a Biological Marker of Inflammation: Short-Term Longitudinal Study” (J Med Internet Res 2023;25:e46309) one error has been noted.

In the originally published manuscript, Reference 1 contained a broken URL.

This URL has been corrected to: https://onlinelibrary.wiley.com/doi/10.1111/j.1083-6101.2007.00393.x [1]

The correction will appear in the online version of the paper on the JMIR Publications website February 1, 2024, together with the publication of this correction notice. Because this was made after submission to PubMed, PubMed Central, and other full-text repositories, the corrected article has also been resubmitted to those repositories.
