# Supplementary material for: Social Media Use and Its Concurrent and Subsequent Relation to a Biological Marker of Inflammation: Short-Term Longitudinal Study
Source: J Med Internet Res. 2023 Dec 8;25:e46309. doi: 10.2196/46309 (PMC10746962; doi:10.2196/46309)
Supplement: Multimedia Appendix 1 [file jmir_v25i1e46309_app1.docx]

**Multimedia Appendix 1.** Social media use data collection using the iOS Screen Time app.

This document contains details on how social media use data were collected and a set of supplementary analyses for the longitudinal effects of SMU on CRP. This document also includes a set of exploratory analyses testing whether our findings are moderated by gender.

Details on collecting social media use using the iOS Screen Time application

To collect the amount of social media use for the week of Phase 1, we contacted participants one week after their initial visit to the lab and asked them to report their SMU in the *past* week, thereby aligning their SMU with the CRP measurement. We chose this method because of the way the iOS Screen Time application works. The Screen Time app provides weekly average use data from Sunday to Sunday. This means that if participants were to report their SMU for the *current* week, they could have weekly average use data based on a different number of days. For example, a participant coming to the laboratory on a Monday would provide a “weekly” average number based on their use from Sunday to Monday; another participant coming to the laboratory on a Friday would provide a “weekly” average number based on their use from Sunday to Friday. Our solution to this issue was to ask participants to report their use in the *past* week by contacting them one week after Phase 1 so that all participants’ weekly average use data are based on their use over one full week (7 days).

The second blood draw was the termination of the study. Accordingly, there was not a survey sent to the participant on the Monday after the blood sample, unlike in Phase 1. Thus, for the cross-sectional analysis at Phase 2, Phase 2 SMU refers to time spent on social media for the entire week (Sunday to Sunday) preceding the week when participants came back to our laboratory to provide their blood for CRP the second time. Because this laboratory session occurred on a weekday, SMU data for the entire week were not available. On average, the CRP measurement preceded Phase 2 SMU by 2.9 days (SD 1.46). Therefore, we used SMU data from the prior week, which we believe should provide the best available proxy for their weekly SMU.

Sensitivity analyses for the longitudinal findings (testing H2)

To further bolster our longitudinal findings, we conducted an additional set of multiple regression analyses with amount of social media use at Phase 1 as a predictor of CRP at Phase 2 while controlling for CRP at Phase 1. The amount of social media use at Phase 1 predicted CRP at Phase 2 in Model 1 (*β* = .28, *P* = .007), Model 2 (*β* = .30, *P* = .005), Model 3 (*β* = .31, *P* = .006), and Model 4 (*β* = .30, *P* = .008), suggesting that the impact of social media use on CRP may occur over time and further supporting our H2. The results of these analyses are detailed in Table S1. See Figure S1 for the scatterplot.

**Table S1.** Coefficients from linear regression models predicting CRP at Phase 2

| Predictor | Model 1 | | | | Model 2 | | | | | Model 3 | | | | | Model 4 | | | | |  |
| --- | --- | --- | --- | --- | --- | --- | --- | --- | --- | --- | --- | --- | --- | --- | --- | --- | --- | --- | --- | --- |
|  | B | SE | *t* | *P* | | B | SE | *t* | *P* | | B | SE | *t* | *P* | | B | SE | *t* | *P* | |
| Gender | .03 | .10 | .27 | .79 | | .04 | .11 | .34 | .73 | | .04 | .11 | .34 | .74 | | .03 | .14 | .18 | .86 | |
| Age | .02 | .01 | 1.22 | .23 | | .02 | .01 | 1.14 | .29 | | .02 | .02 | 1.07 | .29 | | .02 | .02 | 1.03 | .31 | |
| Edu (M) | -.08 | .05 | -1.58 | .12 | | -.07 | .06 | -1.25 | .22 | | -.07 | .06 | -1.24 | .22 | | -.07 | .06 | -1.23 | .23 | |
| Edu (F) | .06 | .05 | 1.25 | .22 | | .05 | .05 | 1.02 | .31 | | .05 | .05 | 1.01 | .32 | | .05 | .05 | 1.00 | .32 | |
| Income | .01 | .02 | .27 | .79 | | .01 | .02 | .41 | .69 | | .01 | .02 | .40 | .69 | | .01 | .02 | .41 | .69 | |
| CRP P1 | .72 | .10 | 7.11 | <.001 | | .66 | .12 | 5.38 | <.001 | | .66 | .14 | 4.75 | <.001 | | .65 | .14 | 4.50 | <.001 | |
| BMI |  |  |  |  | | .01 | .01 | .87 | .39 | | .01 | .01 | .85 | .40 | | .01 | .01 | .85 | .40 | |
| Smoking |  |  |  |  | | .01 | .06 | .03 | .98 | | .01 | .06 | .02 | .98 | | .01 | .06 | .03 | .98 | |
| Alcohol |  |  |  |  | | .02 | .04 | .41 | .69 | | .02 | .04 | .41 | .69 | | .01 | .04 | .38 | .71 | |
| Sit |  |  |  |  | | .04 | .06 | .62 | .54 | | .04 | .06 | .58 | .57 | | .04 | .06 | .58 | .57 | |
| Depres |  |  |  |  | |  |  |  |  | | .01 | .09 | .06 | .96 | | .01 | .09 | .06 | .95 | |
| BirthCon |  |  |  |  | |  |  |  |  | |  |  |  |  | | .02 | .14 | -.17 | .87 | |
| SMU P1 | .01 | .01 | 2.82 | .007 | | .01 | .01 | 2.94 | .005 | | .01 | .01 | 2.88 | .006 | | .01 | .01 | 2.80 | .008 | |
| *R^2^* | .67 | | | | | .68 | | | | | .68 | | | | | .68 | | | | |

^a^Gender: coded with 1 (male) and 2 (female).

^b^Edu (M): highest degree obtained by mother.

^c^Edu (F): highest degree obtained by father.

^d^Income: family annual income.

^e^CRP P1: CRP at Phase 1.

^f^Smoking: # of cigarettes smoked per day in the last 30 days.

^g^Alcohol: frequency of alcohol consumption.

^h^Sit: amount of time spent sitting in the past month.

^i^BirthCon: consumption of birth control medication. BirthCon was coded with 0 (not currently taking birth control medication) and 1 (currently taking birth control medication).

^j^SMU P1: social media use at Phase 1.

^k^B values reflect unstandardized coefficients*.*

^l^*R^2^* values reflect those with social media use in the models.

^m^n=52 due to missing values.

Figure S1. Scatterplot of the relation between SMU (Phase 1) and CRP (Phase 2)


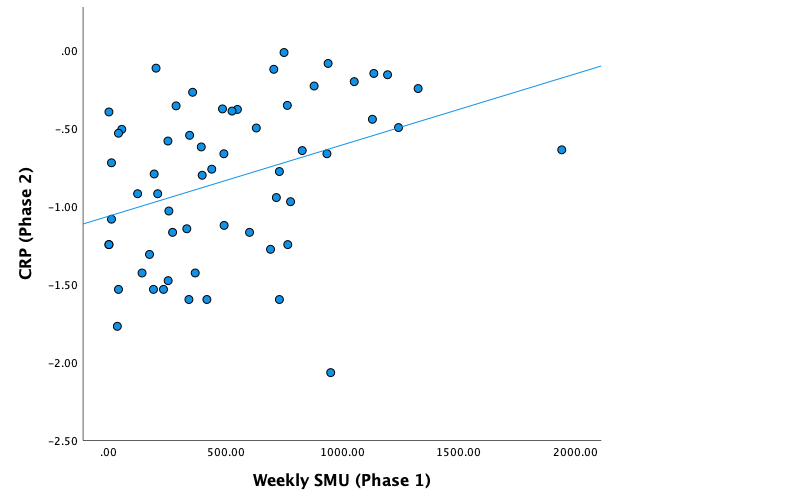


Exploratory analyses with gender as a moderator in the link between SMU and CRP

- Phase 1 SMU predicting Phase 1 CRP with gender as a moderator:
  - Gender X SMU was not significant (*P* = .64).
- Phase 1 SMU predicting Phase 2 CRP with gender as a moderator:
  - Gender X SMU was not significant (*P* = .43)
- Weekly average of SMU from Phase 1 to 2 predicting Phase 2 CRP with gender as a moderator:
  - Gender X SMU use was not significant (*P* = .74)
- Above analyses were tested with Model 4, which provide the most conservative test of our hypotheses (in no other models was Gender X SMU a significant predictor of CRP).
